# Implementation and improvement of policies for building healthy cities in China

**DOI:** 10.3389/fpubh.2024.1399120

**Published:** 2025-01-17

**Authors:** Quansheng Wang, Guoqing Han, Lansong Huang

**Affiliations:** Law School, Shandong University, Weihai, China

**Keywords:** healthy city, health services, healthy environment, healthy culture, healthy life

## Abstract

**Background and purpose:**

With the promotion of the World Health Organization, China has also launched a healthy city construction campaign. However, healthy city construction needs to formulate a series of policies. How can the current healthy city policy in China be further improved to provide a policy basis for healthy city construction?

**Materials and methods:**

Collected here are policy texts from the Central People’s Government and local government of the People’s Republic of China on healthy cities from 2009 to 2023. This paper adopts the policy tool analysis method to design a two-dimensional analysis framework of the “policy tool-construction domain.” There are three types of policy tools: demand-oriented, supply-oriented, and environmental-oriented. The field of healthy city construction is based on five fields defined by WHO: healthy population, health service, healthy environment, healthy culture, and healthy society. The policy text was coded and analyzed by Nvivo software.

**Results:**

According to the coding analysis of policy texts, among the three types of policy tools, supply-oriented policies account for 60.5%, environmental policies account for 29.1%, and demand-oriented policies account for 10.4%. In the five areas of healthy city construction, healthy environment accounted for 23.7%, healthy society accounted for 12.3%, health services accounted for 39.1%, healthy population accounted for 13%, and health culture accounted for 11.9%. From the two-dimensional perspective of policy tools and the field of healthy city construction, the five fields of healthy city construction have different emphases on the application of three different types of policy tools.

**Conclusion:**

Currently, the supply-oriented policy, the demand-oriented policy, and the environmental policy tools are used comprehensively in healthy city construction in China. The proportion of supply-oriented policy is high, which emphasizes the government’s intervention and neglects the participation of individuals and social organizations. The use of policy tools is not balanced in the five different areas of healthy city construction, which to some extent limits the effect of policy implementation and strengthens the overall effect of the healthy city construction policy.

## 1 Introduction

Healthy Cities is an international initiative advocated by the World Health Organization (WHO). Advance the objective of universal health by tackling the escalating difficulties of urbanization, including environmental degradation, deteriorating infrastructure, insufficient public services, and the proliferation of chronic diseases.

The term “healthy city” was first used by the World Health Organization (WHO) in 1984 during the Healthy Toronto 2000 Conference in Toronto, Canada. The conference’s goal was to encourage broad cooperation between different organizations, sectors, and the general public to address health and urban sanitation challenges (1, 2). The inaugural Global Conference on Health Promotion convened in Ottawa in 1986, promulgated a proclamation highlighting that the establishment of a healthy city transcends mere sanitation efforts. This encompasses multiple urban sectors and stakeholders, including governmental policies, community assistance, and individual conduct (3, 4). In 1995, the World Health Organization (WHO) established a clear definition of a healthy city as “a city where the natural and social environments are perpetually improving, enabling individuals to support one another in enjoying life and realizing their full potential through the continuous enhancement of social resources” (5). The Patriotic Public Health Commission of China characterizes a healthy city as an enhanced iteration of a sanitary city. A healthy city enhances natural, social, and sanitary environments while promoting healthy lives through superior urban planning, construction, and management. To address the health requirements of the population and attain the integrated advancement of urban development and human health (6).

Between 1985 and 1986, the WHO European Region initiated the “Healthy Cities Program,” aimed at fostering the establishment of healthy cities. The World Health Organization’s European Region Office initiated the “Healthy Cities Project” in 1986. Consequently, the European Network of Healthy Cities was founded. In 1987, Canada formally initiated the Healthy City Program, encompassing the “Healthy Cities Movement” in Toronto and the “Healthy Cities and Towns Movement” in Quebec. Canada initiated the “Healthy Communities” campaign, reflecting the size and sparsity of its territory, which subsequently encompassed the entire nation (7).

The United States is actively involved in the development of sustainable cities. In 1989, the US Department of Health and Human Services officially adopted the notion of healthy communities and advocated for it on a national scale. Indiana has prioritized the cultivation of local community leadership as the principal technique for implementing the Healthy City Program. The strategy prioritized the execution of the Healthy City Program via local community engagement and development, establishing the “U.S. Model” for the creation of healthy communities (8).

During the late 1980s and early 1990s, as developed nations such as Europe and the United States initiated Healthy Cities programs, Australia, Malaysia, Japan, and New Zealand in the Western Pacific region similarly adopted the initiative. In 1996, Cambodia, Laos, North Korea, Mongolia, and South Korea launched the Healthy City Program. Countries are enhancing urban health by prioritizing healthy city design, enforcing sanitary management legislation, implementing anti-pollution measures, and including all inhabitants in urban sanitation programs. The Healthy City Program has evolved into a global initiative in urban areas worldwide. Since the inception of the Healthy Cities Program, over 2,000 cities across Asia, Africa, Europe, North America, South America, and Australia have participated (9, 10).

The notion of a healthy city was first presented in China during the 1990s, generally coinciding with the creation of national sanitary cities in the late 1980s. In 1993, China formally initiated health city planning operations. In 1994, the Dongcheng District of Beijing and the Jiading District of Shanghai launched a pilot initiative for the development of healthy cities. In 2001, Suzhou became the inaugural Chinese city to announce its involvement in the Healthy Communities Construction Program to the World Health Organization (WHO) and establish a preliminary set of guidelines for healthy communities. Following the Severe Acute Respiratory Syndrome (SARS) epidemic in 2003, the development of healthy cities in China progressed to a phase of comprehensive and significant advancement. The second Global Conference of the Alliance for Healthy Cities occurred in Suzhou in 2006. In 2008, the former Ministry of Health of China presented the “Healthy China 2020 Strategy” and designated 10 cities, including Shanghai, Hangzhou, and Suzhou, as experimental programs. The inaugural WHO Healthy Cities Collaborating Center Network in China was created in Shanghai in 2013. The network consists of 46 groups in Shanghai, Hangzhou, Suzhou, and other cities committed to advocating for healthy lifestyles and preventing chronic diseases.

In 2012, the State Council of China promulgated the “Twelfth Five-Year Plan for Health Development,” signifying the comprehensive initiation of healthy city construction initiatives. In 2015, the Fifth Plenum of the 18th Central Committee of the Communist Party of China resolved to advance the development of health-oriented towns with distinct Chinese attributes. The State Council released “Opinions on Enhancing the Patriotic Public Health Campaign in the New Era.”

In 2016, the national “13th Five-Year Plan” encompassed the advancement of constructing a healthier China. The Patriotic Public Health Commission of China released “Guiding Opinions on the Development of Healthy Cities and Healthy Villages.” In October 2016, the State Council of China released the “Healthy China 2030” Planning Outline, which underscores the establishment of healthy cities and villages as essential measures for constructing a healthier China. In November 2016, the Patriotic Public Health Commission of China initiated the Healthy Cities trial Program, announcing a roster of 38 national trial cities. The “Shanghai Declaration on Promoting Health in the 2030 Agenda for Sustainable Development” was simultaneously issued at the 9th Global Conference on Health Promotion, effectively linking the advancement of healthy cities with sustainable development objectives. In 2017, the National Health and Family Planning Commission of China, the State General Administration of Sports of China, and the All-China Women’s Federation collaboratively released the “Action Program for Healthy Lifestyles of All People (2017–2025)” to advocate for the implementation of a sustainable development model and lifestyle. In 2018, the Patriotic Public Health Commission of China promulgated and enacted the “National Healthy City Evaluation Indicator System (2018 Edition).”

The “Healthy City Project” was a project that highlighted the importance individuals attribute to health by including all sectors of society in cooperative endeavors, encompassing urban design, building, and comprehensive health management. This strategy aims to foster synergies among the population, environment, and society through integrated development to avert diseases and enhance health. The primary objective is to create the healthiest city attainable (11). The building of a healthy city necessitates the collaborative efforts of various stakeholders, including government, businesses, communities, and the populace, alongside good urban administration in all its dimensions. Consequently, the government must implement pertinent policies, efficiently amalgamate human, financial, material, and technological resources, and synchronize the collaborative efforts of all involved parties to accomplish the objective of establishing healthy cities. Policy instruments have been employed throughout many sectors as distinct processes and strategies by individuals to attain public management objectives (8, 12, 13). This study analyzes Chinese national and local government policy papers about healthy cities and assesses policy instruments. The objective is to furnish scientific counsel for improving policies about the advancement of healthy cities.

## 2 Materials and methods

### 2.1 Data sources

This study chose policy documents about the development of healthy cities since the launch of the “Healthy China 2020 Strategy,” introduced by the former Ministry of Health in 2008, as samples. The investigation was performed from January 1, 2008, to September 1, 2023, utilizing the terms “healthy cities” and “healthy towns.” The search duration extended from January 1, 2008, to September 1, 2023. The terms “healthy cities” and “healthy towns” were employed to examine the official websites of Chinese administrative entities at various levels, including the State Council, the Health Commission, provincial governments, and municipal governments with districts, as well as the Database of Laws and Regulations of China. This research develops inclusion and exclusion criteria to assure the accuracy and representativeness of the policy wording.

#### 2.1.1 Inclusion criteria

The first requirement is that the phrase “Healthy City” must be included in the title of the publication. Second, the departments that are responsible for issuing licenses are the State Council of China, the National Health and Family Planning Commission of China, the provincial governments, and the local governments that have districts. To put it another way, the entity that is issuing the document must be a government department.

#### 2.1.2 Exclusion criteria

First, the development of the Healthy City Construction Commission and the Healthy City Evaluation Standard System, both of which are examples of organizational setups and technical requirements at a particular operational level. Second, departments of the government at lower levels transmit papers that have been sent by departments and governments at higher levels. Based on the criteria outlined above, thirteen different policy texts were ultimately incorporated into NVivo 14 for policy analysis (see Table 1).

**Table 1:** Policy texts for building healthy cities in China.

No	Name of the policy text	Publishing subject	Policy text number	Implementation date
1	The resolution of the CPC Nanning Municipal Committee and the Nanning Municipal People’s Government about the establishment of a Healthy City	Nanning Municipal People’s Government	Issued by the Nanning Municipal People’s Government (2009) NO. 26	9 September 2009
2	Shaobing Municipal People’s Government Office on the Implementation of Healthy City Creation	Shaobing Municipal People’s Government	Issued by Shaoxing Municipal People’s Government Office (2013) No. 87	18 June 2013
3	The Zhuhai Municipal People’s Government Office on the Issuance of Opinions Regarding the Creation of Healthy Cities	Zhuhai Municipal People’s Government	Zhuhai Municipal People’s Government Office, 2014, No. 10.	4 April 2014
4	The Implementation of the Anhui Patriotic Health Campaign Committee’s Opinions on the Construction of Healthy Cities, Healthy Villages, and Towns	Anhui Patriotic Health Committee	Anhui Patriotic Health Campaign Committee (2016) No. 45	15 July 2016
5	A Circular of the National Patriotic Health Campaign Committee on the Issuance of Guiding Opinions on the Construction of Healthy Cities, Healthy Villages, and Towns	National Patriotic Health Campaign Committee	Issued by the National Patriotic Health Campaign Committee (2016) No. 5	18 July 2016
6	The People’s Government of Puyang City’s Implementation Opinions on the Construction of a Healthy City	Puyang Municipal People’s Government	Puyang Municipal People’s Government (2016) No. 63	30 August 2016
7	A notice was issued by the General Office of the Zibo Municipal People’s Government to implement opinions regarding the construction of healthy cities, villages, and municipalities.	Zibo Municipal People’s Government	Zibo Municipal People’s Government Office (2017) No. 45	5 April 2017
8	The General Office of the Dalian Municipal People’s Government’s Implementation Opinions on the Further Strengthening of the Construction of Healthy Cities, Healthy Villages, and Towns	Dalian Municipal People’s Government	Issued by the Office of Dalian Municipal People’s Government (No. 54)	14 April 2017
9	The Weifang Municipal People’s Government Office on the Implementation of Opinions on the Construction of Healthy Cities, Healthy Villages, and Towns	Weifang Municipal People’s Government	Weifang Municipal People’s Government Office, (2017). 72	21 July 2017
10	The Opinion of the Longnan Municipal People’s Government on the Implementation of Healthy Cell Construction, Healthy Townships and Villages, and the Healthy City	Longnan Municipal People’s Government	Issued by the Longnan Municipal People’s Government in 2017 (No. 54)	27 August 2017
11	The Panjin Healthy Cities, Healthy Villages, and Town Construction Implementation Program notice has been issued by the Panjin Municipal People’s Government Office.	Panjin Municipal People’s Government	Issued by the Panjin Municipal People’s Government Office (No. 127)	6 November 2017
12	The Office of the People’s Government of the City of Taian has issued a notice regarding the issuance of opinions regarding the implementation of the construction of healthy cities, villages, and towns in Taian City.	Taian Municipal People’s Government	Taian Municipal People’s Government Office (2018) No. 18	27 April 2018
13	Circular of the General Office of the Shanghai Municipal People’s Government: Transmitting the Opinions of the Municipal Health Commission and 14 Other Departments on the Strengthening of Community Health Services in the City to Promote the Development of Healthy Cities	Shanghai Municipal People’s Government	Issued by the Office of the Shanghai Municipal People’s Government (2019) No. 2	16 January 2019

### 2.2 Framework for analysis

The classification of policy instruments developed by Zegveld and Rothwell served as the basis for the construction of a framework for the analysis of policy documents (14) and the two dimensions of healthy city construction. What are the reasons for selecting this specific type of policy classification and the five dimensions of healthy city construction? We contend that the construction of a healthy city should prioritize the enhancement of the natural and social-ecological environment, the improvement of public health, the overall health level of the city, the creation of an attractive and suitable environment for human settlements, and the enhancement of the comprehensive development quality and sustainable development capability of cities. This will facilitate the potential for healthy cities to develop further and become more conducive to entrepreneurship, as well as foster a more harmonious and dynamic economic, social, and ecological environment. Furthermore, this will lead to an improvement in the quality of health and quality of life, enabling the public to enjoy a better way of life as a result of improved health. Accordingly, the aforementioned classification of healthy urban construction policy is compatible with three distinct types of policy: demand-based, supply-based, and environmental-based. Additionally, Chinese scholars concur that the foundation of healthy city construction is comprised of five key elements: health services, a healthy environment, a healthy culture, a healthy society, and healthy people. Furthermore, the “Outline for the Implementation of the Construction of a Healthy China,” as issued by the Chinese government, also encompasses the aforementioned five areas. These form the theoretical basis and research framework of the subject.

**Figure 1:**
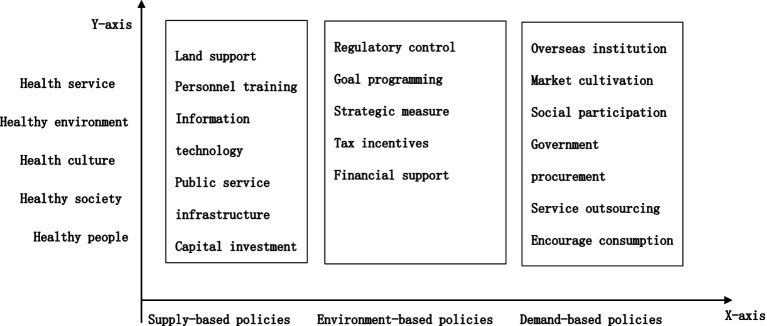
Two-dimensional analysis framework of healthy urban construction in China.

There are three categories into which policy instruments can be classified: environmental, supply, and demand policy. The five main areas of healthy Chinese construction, which include health services, the health environment, the health culture, the health society, and the health population, define the field of healthy city construction. The y-axis represents the realm of healthy city construction, which establishes a two-dimensional analysis framework (see Figure 1).

#### 2.2.1 X dimension: policy instrument dimension

Policy instruments are the methods or modalities employed by the government to accomplish its policy objectives (15). At present, Zegveld and Rothwell’s classifications are the most frequently employed. The policy instruments were categorized into three categories: environmental, supply-based, and demand-based. The classification instrument is more precisely defined and operationalized (16). Consequently, this instrument was designated as the *X*-axis inside the two-dimensional analytical framework of this study. Supply-oriented policy instruments predominantly entail national assistance for the development of healthy cities via cash, knowledge, and technology. The principal objective of policies advocating for the development of healthy cities. Environmental policy instruments foster the development of healthy cities through targeted planning, regulatory frameworks, financial support, and additional mechanisms. Demand-driven policy instruments facilitate the advancement of healthy cities and enhance chances for such growth via market stimulation, government procurement, and service outsourcing. Please see Table 2 for a detailed list of specific policy instruments and their definitions.

**Table 2:** The basic types and meanings of policy tools for healthy urban construction.

Tools type	Tools name	Description of tool meanings
Demand type	Market cultivation	Enhance publicity and promotion efforts, foster and support the advancement of the health industry, and facilitate the establishment of a comprehensive and multi-tiered health service market.
	Government procurement	Government departments engage in the procurement of products from the emerging health industry, both directly and indirectly, to foster its development.
	Services outsourcing	To delegate the development, infrastructure construction, personnel training, and other necessary projects for the establishment of healthy cities to external entities.
	Overseas institutions	Investigate the international market of the burgeoning health sector and facilitate the multifaceted advancement of the health industry.
	Social participation	We will promote the active involvement and support of social entities in the development of healthy cities, integrate resources from diverse social organizations, and create a scenario where the entire population is engaged and participates collectively.
	Encouragement of consumption	To enhance consumption and advance the development of health industries through the encouragement and promotion of emerging health sectors.
Supply type	Personnel training	Facilitate talent support for the development of healthy cities, including talent training and recruitment initiatives.
	Information technology	Develop an intelligent platform for health information, enhance data collection and integration, and facilitate the interconnection and sharing of health information; advance public health communication initiatives, and establish new media channels for health promotion in the development of healthy cities.
	Public service	Implementing government functions and enhancing its active role through the establishment of a public service platform, the improvement of the service guarantee system, and the formulation of specific measures for the development of healthy cities.
	Infrastructure	This encompasses the development of infrastructure, including roads, sanitation systems, electricity provision, communication networks, and firefighting services. Examples include urban sewage and waste treatment facilities, as well as sanitary public toilets designed for safety and hygiene.
	Land support	To develop healthy urban environments through the provision of land use indicators, the revitalization of land resources, and the optimization of land use approval processes.
	Investment	The government will facilitate the efficient development of healthy cities through financial investment and subsidies.
Environmental type	Regulatory control	The implementation of diverse laws and regulations aims to enhance the quality and safety supervision system for agricultural products, reinforce oversight of food and drugs, and mitigate the incidence of food and drug safety events.
	Goal programming	The government develops a healthy city plan by analyzing the primary factors influencing health in the current context.
	Tax preferences	Implement preferential policies for the development of healthy cities via tax reductions and fee waivers.
	Strategic measures	Implementing diverse environmental remediation activities, enhancing the ecological protection system, and refining the health management system and mechanisms are essential for achieving policy objectives effectively.
	Financial support	Facilitate credit and guarantees for the construction of diverse facilities; ease financial constraints within the health sector, among other measures.

#### 2.2.2 Y dimension: healthy city building areas

The World Health Organization (WHO) advises that every healthy city aims to attain the following objectives: establishing a friendly environment that promotes health. Improve the quality of life for the populace. Guaranteeing the fulfillment of fundamental sanitary requirements for the populace. Improve accessibility to sanitary services. The objective of healthy city development is to progressively achieve an urban model that is economically thriving, socially integrated, environmentally pristine and esthetically pleasing, culturally vibrant, secure, joyful, and habitable (17, 18). Healthy city strategies integrate social, economic, geographical, ecological, infrastructure, and various other urban systems with complex policy substance. To comprehend healthy city policy tools, one must analyze primary assessment indicators. In 2018, they were incorporated into the “National Healthy City Evaluation Indicator System” by the Patriotic Public Health Commission of China. This paper presents a Y-dimensional method for the development of healthy cities. Classify healthy city policies into five subsystems: a healthy environment, a healthy society, health services, healthy individuals, and a healthy culture (19), unidimensional policy instruments can reflect the primary methods and approaches of policy actions but may not fully demonstrate policy objectives and operational characteristics. Based on the research findings of experts and scholars on healthy cities both domestically and internationally (3, 20, 21), this paper establishes a two-dimensional analytical framework of “X dimension (policy instruments) – Y dimension (healthy city construction field)” to examine the policy documents related to healthy cities. The research and policy texts of the experts are combined to construct this framework (see Figure 1).

### 2.3 Research methodology and policy text coding

NVivo is a software for text content analysis. The phrase “content analysis” refers to a methodological approach employed in the scientific community to investigate the fundamental nature of a phenomenon by analyzing it via its framework. The proposed method enables the reproduction of policy text content while extrapolating the outcomes. The thirteen policy documents about healthy cities in China were categorized by “policy number (Title 1–Title 2).” The idea of “non-disaggregation” establishes the minimal unit that characterizes the subject of measurement in the content analysis methodology. This concept guarantees the comprehensiveness of the meaning. A paragraph conveying a singular meaning serves as the analytical unit. In a text with several levels of meaning that may be segmented into several sentences, the sentence functions as the analytical unit (22, 23). The designation 13–4–5 indicates that the thirteenth policy document is titled “Circular of the General Office of the Shanghai Municipal People’s Government Transmitting the Opinions of the Municipal Health Commission and 14 Other Departments on Strengthening Community Health Services in the City to Promote the Development of Healthy Cities.” This chapter comprises 246 units of policy content analysis in the fifth paragraph under the fourth-level topic. We assess each analytical unit in the original text using contextual analysis to generate clear classifications for policy instruments and domains associated with the development of healthy cities. The units are thereafter categorized according to their distinct meanings. The application of policy instruments to foster the advancement of healthy cities is illustrated by quantitative data. To validate the coding’s reliability, two researchers independently coded the data, followed by a final consistency assessment of the coding outcomes. The test produced a kappa coefficient of 0.905, signifying a substantial level of consistency in the coding outcomes. Owing to spatial limitations, only a selection of coding results is presented (see Table 3).

**Table 3:** China healthy urban construction policy text coding table.

No	Planning name	Content analysis unit for policy texts	Code
1	The decision of the CPC Nanning Municipal Committee and Nanning Municipal People’s Government on Building a Healthy City	3 Main tasks and measures for building healthy cities 3.1 Strengthen the construction and management of urban public facilities and vigorously create a healthy environment. 3.1.1 Strengthen infrastructure construction. By the requirements of a high-starting point plan, high-intensity investment, high-standard construction, and high-efficiency management, large projects improve the urban and rural infrastructure network and meet the needs of the people’s healthy life. 3.2 Strengthening disease control and health services and vigorously improving health services 3.2.1 Improve the disease prevention and control system. Accelerating the capital construction of specialized institutions for disease prevention and control at all levels, improving equipment and facilities, and raising the quality of professional teams. 3.3 Strengthening health education and management, and vigorously cultivating a healthy population 3.3.1 The proliferation of preventive healthcare immunizations is a primary goal. According to the State Council’s Regulations on Vaccine Circulation and Immunization Planning Administration, the Government has taken on increased responsibility for developing and refining the long-term management framework for immunization planning, ensuring effective preventive healthcare and health promotion across all demographics, and improving the quality and management of immunization planning efforts. The program targets rural and migratory populations, progressively broadening the range of pediatric immunizations within the immunization initiative, guaranteeing the execution of preventative vaccination efforts, and creating a solid platform for health promotion.	1-3-1-1	1-3-2-1	1-3-3-1
	……	……	……
13	Circular of the General Office of the Shanghai Municipal People’s Government transmitting the Opinions of the Municipal Health Commission and Fourteen Other Departments on Strengthening Community Health Services in the City and Promoting the Development of Healthy Cities.	4 Main tasks 4.2 Strengthening community medical and health services 4.2.1 Creating health accounts focused on residents. Enhance the electronic health records of residents in the city by progressively incorporating the records of community residents’ visits to diverse medical institutions, service records from various social health management entities, student health records, physical fitness monitoring data, and residents’ self-health monitoring records. Achieve the interconnectivity of information among health service institutions. 4.3 Strengthening community services for healthy aging 4.3.1 Developing community care and home care services. Continuously expanding the supply of older adults care services, guiding community care organizations to develop in the direction of embeddedness and multi-functionality. 4.4 Strengthening community health promotion and education 4.4.1. Establishing a multi-level health publicity and education system. Relying on the three-tiered network of community colleges, community schools, and village learning points, health education is being carried out to advocate a healthy lifestyle.	13-4-2-1		13-4-3-1	13-4-4-1

Coding rules: The policy tools and the policy goals used by a specific measure should be coded as the classification rules, and one measure corresponds to one policy tool and one policy goal. Take a specific measure in the first document as an example: Improve the environmental sanitation infrastructure. We will strengthen the construction of urban sewage and garbage treatment facilities, and gradually realize the complete collection and treatment of urban sewage, the centralized disposal of urban medical waste, and the reduction, resource, and harmless treatment of urban household garbage. We will accelerate the construction of urban public toilets, and form an urban public toilet service system with a reasonable layout, sufficient quantity, perfect facilities, and standardized management. We will promote dust-reducing and low-dust cleaning operations, expand the scope of mechanized cleaning and cleaning operations, and improve the construction and management level of urban municipal public facilities. The policy tool used in this measure is supply-based infrastructure, and the policy objective it aims to achieve is a healthy environment. The presentation rule of coding: Taking the title of the policy text itself as the reference basis, according to the progressive relations of the first-level heading, the second-level heading, and the third-level heading, the coding classification is made for searching. Taking the above-mentioned measure as an example, it is located in the second secondary title in the third level title in the first document and is coded as 1-3-2. When coding, as far as possible, one paragraph is a code, but if there are several equally important different policy tools in a paragraph, it is divided by sentence.

The presentation rule of coding: Taking the title of the policy text itself as the reference basis, according to the progressive relations of the first-level heading, the second-level heading, and the third-level heading, the coding classification is made for searching. Taking the above-mentioned measure as an example, it is located in the second secondary title in the third level title in the first document and is coded as 1–3–2. When coding, as far as possible, one paragraph is a code, but if there are several equally important different policy tools in a paragraph, it is divided by sentence.

## 3 Results

### 3.1 X dimension: basic information on policy instruments

The results of the utilization of policy instruments were derived from the categorization and generalization data based on the Healthy Cities policy content analysis unit coding form (see Table 4). The existing planning policies for the Healthy China initiative predominantly employ supply-based, environment-based, and demand-based strategies, with utilization rates of 60.5, 29.1, and 10.4%, respectively. The three types of policy instruments demonstrate notable disparities in their utilization frequency, with supply-based policies being employed more frequently than demand-based ones.

**Table 4:** Distribution table of basic policy tools in China’s healthy urban construction policy texts.

Types of policy instruments	Tool name	Policy text number	Count	Percent
Demand type	Market cultivation	2–2–3–6;3–2–5–5;3–3–4–4;7–4–2–3–6;9–3–2–3;10–3–1–2–6	6	10.4%
	Government procurement	N/A	N/A	
	Service outsourcing	N/A	N/A	
	Overseas institution	N/A	N/A	
	Social participation	1–3–3–4;1–3–4–3;2–3–4;2–4–3;3–4–3;4–6–3;5–4–4;6–5–3;7–4–5–2;8–5–3;8–5–4;…	21	
	Encourage consumption	N/A	N/A	
Supply type	Personnel training	1–3–2–3;7–5–5;9–2–3–5;10–3–1–2–6;11–4–4;13–4–3–3	6	60.5%
	Information technology	2–3–10;4–5–1–2;7–4–7;7–4–2–1;9–3–2–1; 13–4–2–1;13–4–7–1;…	10	
	Public service	1–3–1–2;1–3–1–3;1–3–1–4;1–3–3–1;1–3–3–2;2–2–2;2–2–3–3;2–2–4;…	115	
	infrastructure	1–3–1–1;1–3–2–1;2–2–1;3–3–1–2;3–3–2–2;3–3–2–3;3–3–3–1–1;3–3–3–2–1;4–5–1–3;…	25	
	Land support	N/A	N/A	
	Capital investment	5–4–2;8–5–2;9–7–2;10–4–2;11–4–2;12–6–4;13–5–4	7	
Environmental type	Regulatory control	1–3–4–4;2–2–3;3–3–1–5;4–4–2–7;4–5–1–6;5–3–11;6–3–6;9–3–6;10–3–1–3–3;…	13	29.1%
	Goal programming	1–2-2;2–1-3;3–1-3;3–4-2;4–6-2;4–3;5–2-1;6–5-2;6–1-3;…	18	
	Tax incentives	N/A	N/A	
	Strategic measure	1–3–2-1;1–3–2-2;1–3–2-4;2–3-2;3–2-1;3–3–1-4;3–3–2-4;3–3–3-1-2;4–5–1-5;…	37	
a	Financial support	N/A	N/A	

N/A means that this policy instrument is not used in the policy text.

Regarding specific content, alongside the “land support” policy instrument, the remaining five supply-based policy instruments—personnel training, information technology, public services, infrastructure, and capital investment—have all been executed. Among the 163 policy instruments analyzed, 115 (70.6%) are classified as public service instruments, while 25 (15.3%) are categorized as infrastructure instruments. Information technology and financial inputs accounted for 6 and 4%, respectively, while human resource development comprises 3.6%. This indicates that in the supply-oriented policy for healthy city growth, the government prioritizes public services and infrastructure enhancement. The oversight of policy tools like “land support” and “personnel training” in supply-oriented policies can considerably restrict the quality and extent of healthy city growth.

Of the five environmental policy instruments, financial support and tax incentives are underutilized, whereas regulatory control, goal programming, and strategic measures are effectively employed. Among the 68 units analyzed for policy content, strategic measures comprised 37 (54.4%), goal programming constituted 18 (26.5%), and Regulatory control represented 13 (19.1%). Current endeavors to promote healthy cities in China prioritize strategic strategies, targeted planning, and legal regulatory instruments while neglecting financial support and tax incentive policies.

Among the six distinct instrumental methods of demand-based strategies, government procurement, service outsourcing, overseas institutions, and encouraging consumption are not employed. Only two instruments have been executed: social participation and market incubation. Among the 27 units of policy content analysis, 21 (77.8%) pertained to social participation, whereas 6 (22.2%) were associated with market formation. Among the supply-based, environmental, and demand-based policy categories, demand-based policies employ fewer instruments, with only a limited percentage being implemented. Our healthy city policy does not prioritize a demand-driven policy framework.

### 3.2 Y dimension: basic information about the healthy city construction field

The coding table for the content analysis unit of the Healthy City Policy facilitated the classification, summarization, and statistical analysis of the distribution of construction areas within China’s Healthy City Policy. Out of 253 policy content analysis units, 60 (23.7%) pertain to healthy environment construction, 31 (12.3%) to healthy society construction, 99 (39.1%) to health services construction, 33 (13%) to healthy population construction, and 30 (11.9%) to the culture of health construction, across the five principal domains of healthy city development. Our country has concentrated on five distinct domains of healthy city development. The establishment of a healthy environment constituted a marginally greater proportion than the other four specialized domains.

### 3.3 X-Y dimension: policy instruments for healthy city construction: two-dimensional basics

The results of the two-dimensional study of healthy city policies X-Y are generated from the examination of policy instruments in the X dimension and the incorporation of healthy city building areas in the Y dimension (see Table 5). Three policy tools concentrate on each of the five overarching domains of healthy city development. In health service construction, 99 units were utilized for policy content analysis: 57 supply-oriented policies (57.6%), 23 environmental policies (23.2%), and 19 demand-oriented policies (19.2%). In health care, policymakers have primarily supported supply-oriented approaches over those based on environmental and demand considerations. 60 units are offered for the analysis of policy content in the context of creating a healthy environment. Of them, 31 (51.7%) are supply-oriented policies, whereas 29 (48.3%) are environmental policies. 30 policy analysis units are available for the creation of health culture. Of these, 25 (83.3%) are supply-oriented policies, 3 (10%) are environmental policies, and 2 (6.7%) are demand-oriented policies. There are 31 policy analysis units dedicated to fostering a healthy society. Of them, 19 (61.3%) are supply-oriented policies, 12 (38.7%) are environmental policies, and there are no demand-oriented policies adopted. Within the domain of public health promotion, there exist 33 policy analysis units: 31 supply-oriented policies (94%), 1 environment-oriented policy (3%), and 1 demand-oriented policy (3%).

**Table 5:** Results of X-Y two-dimensional analysis of Chinese Healthy Urban Construction Policy texts.

Types of policy instruments	Concrete tool	Healthy people	Healthy society	Health culture	Healthy environment	Health service
Supply-based policies	Personnel training	0	0	0	0	6
	Information technology	0	0	1	0	9
	Public service	30	19	24	8	34
	infrastructure	1	0	0	23	1
	Land support	0	0	0	0	0
	Capital investment	0	0	0	0	7
Environment-based policies	Regulatory control	0	12	0	0	1
	Goal programming	0	0	0	0	18
	Tax incentives	0	0	0	0	0
	Strategic measure	1	0	3	29	4
	Financial support	0	0	0	0	0
Demand-based policies	Market cultivation	1	0	1	0	4
	Government procurement	0	0	0	0	0
	Service outsourcing	0	0	0	0	0
	Overseas institution	0	0	0	0	0

In total, three policy instruments were recognized throughout the five domains of healthy city development: supply-side policies, environmental policies, and demand-side policies. Nonetheless, supply-oriented policies are employed more frequently, while demand-oriented policies are somewhat less prevalent.

## 4 Discussion

### 4.1 The utilization of policy instruments exhibits considerable variability, and their structure is disproportionately distributed across various domains in the development of a healthy city

The development of healthy cities typically prioritizes supply-oriented policy instruments, while inadequately employing environment- and demand-oriented policy instruments. Categorical statistical analysis reveals that supply-oriented policy instruments with substantial government engagement are predominantly employed in initiatives focused on cultivating healthy cities. They offered several policies and actions across five distinct domains: a healthy environment, a healthy society, health services, healthy individuals, and a healthy culture. Nevertheless, demand-driven policy instruments that emphasize the independence of various social agents and amalgamate governmental power with market dynamics have not been completely utilized. Consequently, they are unable to stimulate the passion of varied participants and market dynamics (24). The implementation of this policy instrument is contingent upon compliance with a certain set of regulations. Advocating for the government’s perspective is essential when implementing a new policy. Moreover, if the policy garners community acceptance, it will transition from a supply-centric approach to one that prioritizes environmental and demand considerations. From a policy implementation standpoint, an optimal policy achieves equilibrium among several policy types. Consequently, each policy must be formulated to enhance the others to guarantee the attainment of the intended outcome (25).

### 4.2 The focus on healthy city development differs among urban regions

The World Health Organization (WHO) and governmental proposals for developing healthier cities have led to considerable emphasis from both central and local governments on the establishment of healthy urban environments. Relevant policy documents and action plans have been issued. The initiative and creativity of local governments in formulating policies for healthy cities are comparatively limited. Many local healthy city policies appear to be derived from the frameworks established by the central government or higher-level state entities. Their efforts have not prioritized the establishment of policies and administrative programs suitable for fostering healthy cities, tailored to the specific realities of their local contexts. Numerous entities comply with the directives established by higher-level authorities and perceive policy formulation as a political achievement, frequently overlooking the implications of its execution (26, 27).

An optimal environment is essential for the protection of human health. A statistical examination of the utilization of policy tools indicates that, at the national level, there is an emphasis on developing and advocating for healthy environments as a priority topic. This also indicates that healthy cities embody an enhanced iteration of the concept of sanitary cities (28, 29). Nevertheless, the Healthy City Program’s development is not only reliant on establishing a healthy environment to attain the objective of being the city with the greatest health standards. It necessitates extensive advancement across multiple domains.

## 5 Conclusion and policy recommendations

The World Health Organization’s Healthy City Program is founded on two essential principles (30). Initially, we assess the condition of the city. Urbanization is a dominant tendency in the global advancement of human society. It is a fundamental necessity and an unavoidable result of the progress of societal productive forces. The evolution of urban areas has significantly enhanced human life and labor, facilitating the swift progression of the global economy. Approximately 50% of the global population resides in metropolitan, constructed environments. The swift advancement of urban growth, particularly in industrialized cities, has resulted in several social, health, and ecological issues. Social issues including elevated population density, traffic congestion, housing limitations, contaminated drinking water and food resources, deteriorating ecosystems, and violence are emerging as significant dangers to human health. The second concept pertains to a healthy city. Cities are not merely economic entities pursuing efficiency in expansion; they should also serve as optimal environments that promote human health. Cities ought to be perceived as dynamic, evolving entities. This is a contemporary prerequisite for the existence and advancement of urban areas.

“Health,” in a restricted sense, pertains solely to an individual’s physical well-being. The notion of “health” within a healthy city is extensive, grounded in the two pillars of the Healthy City Program, the World Health Organization’s 1984 delineation of the 11 characteristics of a healthy city, and the stipulations of the 10 criteria for a healthy city established in 1996. A healthy city is a cohesive entity that includes healthy individuals, a healthy environment, and a healthy community (31, 32). China underscores the importance of establishing a robust China ought to endorse the principle of “big health,” execute the strategy of “integrating health into all policies,” adhere to the doctrine of “building and sharing,” and leverage the responsibilities of the government, various departments, society, and individuals to collaboratively tackle health challenges arising from urbanization. The development of a healthy city can be delineated into five primary domains: a healthy environment, a healthy population, a healthy society, a healthy culture, and health services, constituting an integrated and cohesive entity.

To advance the development of healthy cities, it is essential to integrate supply-oriented, environment-oriented, and demand-oriented policies. Various policy instruments can be employed in a coordinated fashion to foster the development of healthy local cities, taking into account local conditions. Consequently, the development of healthy cities in China necessitates enhancement through the following methods.

### 5.1 Swiftly modifying the ratio of policy instruments

The development of healthy cities is a policy execution process that encompasses all tiers of governance. During the preliminary phase of building, owing to inadequate hardware infrastructure and the limited knowledge among all social stakeholders, it is essential to employ top-down, supply-oriented policy instruments. Nonetheless, the establishment of a healthy city necessitates a grassroots policy implementation process, and dependence simply on governmental authorities is inadequate. The establishment of a healthy city necessitates the collaborative engagement and advocacy of all individuals due to the existence of various social entities (33). Prolonged use of coercive programs by governments frequently results in diminished efficacy and exacerbated adverse consequences. Every policy instrument possesses distinct properties. Governments must consider all three categories of policy instruments and should not overlook the selection of demand-oriented policy instruments. Demand-driven policy instruments can act as a framework for fostering public engagement (1, 34, 35).

### 5.2 Implement policy instruments in a comprehensive and interdisciplinary approach

The Chinese government has consistently underscored the significance of environmental cleanliness and management. In 1953, China launched a comprehensive health campaign to effectively tackle public health issues and guarantee sufficient hygienic conditions. The government has spearheaded this patriotic initiative. Collaborated with all sectors and engaged the entire community. In the last 70 years, the patriotic health campaign has concentrated on enhancing environmental cleanliness, expanding sanitary toilet access, ensuring safe drinking water, and improving health literacy among the populace. The patriotic health campaign successfully mitigated the prevalence of infectious diseases and enhanced public health. The establishment of a healthy city is essential for fostering a healthy China, a vital component in promoting new urbanization, and an important foundation for the contemporary patriotic health campaign (36, 37). The creation of a healthy city encompasses diverse public policies across numerous sectors and is segmented into several construction phases. This approach necessitates the extensive and multidisciplinary application of policy instruments and the collaboration of diverse social stakeholders. The development of Healthy China is progressively transitioning from a focus on environmental health management to an emphasis on holistic health promotion. The focus has transitioned from the initial emphasis on constructing a singular, localized health environment to a model of integrated social health management including the full spectrum of a healthy environment, society, health services, individuals, and culture (38–40).

### 5.3 Policies are specific and targeted

Multiple regions in China are advancing the initiative for a healthy China in alignment with the central government’s guidance. The foundation for constructing healthy cities differs based on location and environment. Local governments should customize their strategies to local situations to avoid duplicating policies. Nonetheless, the current foundations and settings for healthy cities differ from one location to another. Local governments should tailor their programs to specific conditions to prevent redundancy. Local governments must select among supply-oriented, environmental, and demand-oriented strategies to prioritize the five domains of healthy cities. While the national government prioritizes supply-side policies, it is crucial to stress environmental policies and formulate demand-side strategies at the local level. Simultaneously, it is vital to modify the application of particular policy instruments based on the type of policy. Support for talent development, land assistance, and information technology can be enhanced through supply-oriented policy instruments. The demand-driven policy instruments aggressively seek government procurement assistance, outsourcing services, forming offshore entities, offering consumption incentives, and encouraging social engagement. This fosters the development of synergies for creating healthy cities (41–43).

### 5.4 The system appraisal of the implementation of the healthy city construction policy should continue, with the results of this appraisal forming the basis for further improvements to the policy content

The Chinese patriotic health movement originated in the 1980s and has demonstrated a favorable response to the World Health Organization’s Healthy City construction program since the beginning of this century. To illustrate, the city of Shanghai has augmented its investment in urban environmental construction, completed the construction of the urban park system, completed the tertiary hospital system, gradually popularized the family doctor system, and achieved a high degree of satisfaction with health services (44, 45). Furthermore, it has actively popularized health education, thereby realizing the Trinity of a Healthy City, healthy community, and healthy town (46). This provides an exemplar of healthy city construction activities. It is important to note that the efficacy of healthy city construction activities varies depending on the context. A comprehensive evaluation is essential to identify areas for improvement and to develop strategies to address these shortcomings (47, 48). Promoting the dissemination of health education, enhancing food safety supervision, strengthening public health emergency warning and prevention mechanisms, and improving the medical classification system are key areas for consideration (49). It is essential to advocate a problem-oriented approach and actively improve the policy tools and content of healthy cities, thereby further promoting the level and quality of our healthy city construction (50). This approach should be based on the local reality.

### 5.5 Constructing an indicator system for building healthy cities and strengthening policy synergy

Building a healthy city, setting up a healthy city indicator system, and carrying out an evaluation of its effectiveness are important paths to promote the construction of a healthy city (51, 52). Since the promotion of the Healthy Cities Project, some scholars have begun to research the Healthy Cities Indicator System (53–55). In 2018, the National Office of the Patriotic Health Movement in China conducted research and developed the National Healthy Cities Evaluation Indicator System. This system was designed to align closely with the objectives and tasks outlined in China’s Healthy City construction initiative. It aims to guide cities in improving the natural environment, social environment, and health services, promoting the comprehensive popularization of healthy lifestyles, meeting the health needs of residents, and facilitating the coordinated development of city construction and people’s health. The indicator system consists of 5 first-level indicators, 20 s-level indicators, and 42 third-level indicators, which can objectively reflect the overall progress of the construction of healthy cities in different regions. The indicator system also gives the definition, calculation method, caliber range, source department, and other information of each indicator to ensure that the data collection of healthy city evaluation can be carried out by uniform standards. Level 1 indicators correspond to the five areas of “Healthy Environment,” “Healthy Society,” “Healthy Services,” “Healthy People” and “Healthy Culture,” “Healthy Environment,” “Healthy Society,” “Healthy Services,” “Healthy Population” and “Healthy Culture,” while the secondary and tertiary indicators focus on the main health problems and their influencing factors in the development of Chinese cities (52, 56). Since 2018, the National Patriotic Health Campaign Office has engaged the services of a third-party professional institution to conduct annual evaluations of all national health cities across the country. The evaluations entail the analysis and assessment of the progress of healthy city construction in each city, the identification of areas for improvement, the promotion of continuous quality improvement in the work of healthy city construction, and the advancement of the positive development of healthy city construction (50, 57).

At the same time, it is important to pay attention to policy synergies in the process of formulating and implementing public policies for the development of healthy cities that are constantly being followed up. Firstly, it is important to note that local Healthy City construction is based on the Healthy China 2030 Planning Outline and the central Healthy City policy, and is aligned with the central policy (58). Secondly, it is essential to recognize the potential for synergy among local healthy cities, which may face different specific situations, factors affecting healthy cities, and progress in the construction of healthy cities. The model cannot be replicated, but there is an opportunity to learn from each other’s advanced experiences, based on the local construction of healthy cities (59). Thirdly, there is a synergy between policy tools and policy content. The deployment of different policy instruments is associated with varying degrees of efficacy, and the timing of their utilization is crucial for optimizing the overall impact of policy implementation (8). Fourthly, the process of policy formulation should be aligned with the practical implementation of policies. Policies must evolve by the requirements of practice, while practice also informs the necessity for policy (60). The formulation of policy and the practice of policy should be developed in a coordinated manner to facilitate the comprehensive advancement of all areas and aspects of healthy city construction.

Of course, this study possesses limitations that necessitate additional inquiry. The classification of policy tool kinds was performed according to the software specifications of the coding unit to guarantee precision and impartiality. Nonetheless, the understanding of the content and coding may be erroneous. This could result in code mistakes. The impact of precisely articulated policy recommendations on policy efficiency remains ambiguous. This necessitates more verification, which was omitted from this subject.

## Data Availability

The original contributions presented in the study are included in the article/supplementary material, further inquiries can be directed to the corresponding author.
