# Erratum: Implementation and improvement of policies for building healthy cities in China

**DOI:** 10.3389/fpubh.2025.1565076

**Published:** 2025-02-06

**Authors:** Frontiers Production Office

**Affiliations:** Frontiers Media SA, Lausanne, Switzerland

**Keywords:** healthy city, health services, healthy environment, healthy culture, healthy life

Due to a production error, Guoqing Han was not included as an author in the published article. The final list of authors is: Quansheng Wang, Guoqing Han, Lansong Huang. The corrected Author Contributions Statement appears below.

